# Sharing regional capacity in deceased donor kidney transplantation: experience from a regional collaborative in a metropolitan area

**DOI:** 10.1093/ckj/sfae368

**Published:** 2024-11-30

**Authors:** Tamara Wanigasekera, Isaac Kim, Hannah Maple, Ashish Massey, Maria Kiliaris, Sharmistha Das, Rafez Ahmed, Ahmed Malik, David Game, Abbas Ghazanfar, Nizam Mamode, Ismail Mohamed, Reza Motallebzadeh, Jonathon Olsburgh, Joyce Popoola, Ravindra Rajakariar, Lisa Silas, Michelle Willicombe, Frank J M F Dor, Gareth Jones

**Affiliations:** University College London Department of Renal Medicine, Royal Free London NHS Foundation Trust, London, United Kingdom; Imperial College Renal and Transplant Centre, Hammersmith Hospital, Imperial College Healthcare NHS Trust, London, United Kingdom; Department of Transplantation, Guy's Hospital, Guy's and St Thomas’ NHS Foundation Trust, London, United Kingdom; Department of Transplantation, Guy's Hospital, Guy's and St Thomas’ NHS Foundation Trust, London, United Kingdom; University College London Department of Renal Medicine, Royal Free London NHS Foundation Trust, London, United Kingdom; Imperial College Renal and Transplant Centre, Hammersmith Hospital, Imperial College Healthcare NHS Trust, London, United Kingdom; Department of Renal Medicine and Transplantation, The Royal London Hospital, Barts Health NHS Trust, London, United Kingdom; Department of Renal Medicine and Transplantation, St. George's University Hospitals NHS Foundation Trust, London, United Kingdom; Department of Transplantation, Guy's Hospital, Guy's and St Thomas’ NHS Foundation Trust, London, United Kingdom; Department of Renal Medicine and Transplantation, St. George's University Hospitals NHS Foundation Trust, London, United Kingdom; Department of Transplantation, Guy's Hospital, Guy's and St Thomas’ NHS Foundation Trust, London, United Kingdom; Department of Renal Medicine and Transplantation, The Royal London Hospital, Barts Health NHS Trust, London, United Kingdom; University College London Department of Surgery, Royal Free London NHS Foundation Trust, London, United Kingdom; Department of Transplantation, Guy's Hospital, Guy's and St Thomas’ NHS Foundation Trust, London, United Kingdom; Department of Renal Medicine and Transplantation, St. George's University Hospitals NHS Foundation Trust, London, United Kingdom; Department of Renal Medicine and Transplantation, The Royal London Hospital, Barts Health NHS Trust, London, United Kingdom; Department of Transplantation, Guy's Hospital, Guy's and St Thomas’ NHS Foundation Trust, London, United Kingdom; Imperial College Renal and Transplant Centre, Hammersmith Hospital, Imperial College Healthcare NHS Trust, London, United Kingdom; Centre for Inflammatory Disease, Department of Immunology and Inflammation, Imperial College London, Hammersmith Campus, London, United Kingdom; Imperial College Renal and Transplant Centre, Hammersmith Hospital, Imperial College Healthcare NHS Trust, London, United Kingdom; Department of Surgery and Cancer, Imperial College London, London, United Kingdom; University College London Department of Renal Medicine, Royal Free London NHS Foundation Trust, London, United Kingdom

**Keywords:** collaboration, deceased donation, equity, kidney transplantation, mutual aid

## Abstract

**Background:**

Access to deceased donor kidney transplantation may be restricted in the event of resource limitation induced by extreme peaks in activity or local major incidents, which exceed centre capacity. An organ-sharing protocol was developed by the five London transplant units in 2019 to establish a system for safe transfer of organs and recipients between five regional kidney transplant units. We describe the activity and outcomes over the initial 20-month period.

**Methods:**

National data on kidney transplants performed via the collaborative scheme were obtained from National Health Service Blood and Transplant. Outcomes data was collected locally and analysed.

**Results:**

Sixteen recipients were transplanted between November 2020 and July 2022. The reasons for referral were theatre capacity and an information technology systems failure. Donor kidneys were from 10 brainstem death donors (62.5%) and six circulatory death donors (37.5%). Half of the donors fulfilled standard criteria. Twelve patients (75%) were first transplant recipients. Three (18.75%) were highly sensitized (calculated reaction frequency ≥85%). Three (18.75%) patients required arterial reconstruction. Seven patients (43.75%) had delayed graft function. Median creatinine at 12 months post-transplantation was 134 µmol/L. The median length of stay was 7.5 days. Three recipients (18.75%) died within the first year, two from SARS-CoV-2 infection.

**Conclusions:**

This unique organ sharing collaborative scheme involving five hospitals in London enabled 16 transplants to proceed which otherwise would not have occurred. Although initially established for low-risk donors and recipients, the scheme has evolved to enable transplantation for a wide variety of recipients of varying complexity.

## Graphical Abstract

**Graphical Abstract:**
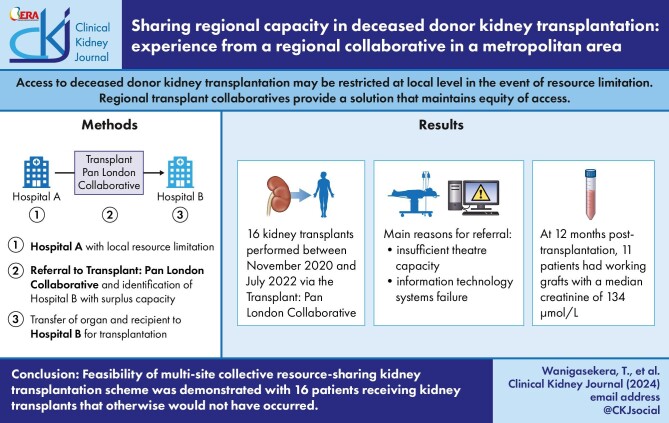

KEY LEARNING POINTS **What was known:**

- Extreme peaks in activity or local major incidents can lead to centre capacity being exceeded and thereby limiting access to kidney transplantation for named patient kidney offers.
- A previous pan-London audit identified 4.6% of declined deceased donor organ offers were due to this reason.

**This study adds:**

- Demonstrates the feasibility of a multi-site collective resource sharing kidney transplantation scheme that allows the provision of mutual aid and restoration of equity of access to transplantation.

**Potential impact:**

- Provide a framework by which similar schemes may be established nationally and internationally.
- Such a collaborative paves the way for amalgamation of best practices and research.

## INTRODUCTION

The offer of deceased donor kidneys for transplantation rarely happens in a linear and orderly fashion. Periods of reduced activity are often followed by multiple simultaneous kidney transplant offers, which can exceed the capacity of transplanting hospitals to implant kidneys in a timely manner. Modern hospitals with multiple specialities competing for access to emergency theatres can put strain on local resources, while major incidents such as mass trauma, information technology (IT) failure and ransomware attacks can put further restrictions on an already constrained system. When unpredictable resource limitation occurs, a transplant may not be able to proceed within the patient's designated hospital and the organ may have to be re-allocated to a different patient in a different hospital, denying access to transplantation for the intended recipient.

In the UK, all deceased donors are allocated on a national basis, according to an agreed algorithm [1]. Organ retrieval is performed by the National Organ Retrieval Service (NORS) and in the majority of the cases these procedures are performed by a team independent of the intended recipient's medical team [2].

The five London transplant units performed 671 deceased donor transplants in the 2022–23 financial year, which accounts for just under 30% of the UK's total deceased donor activity that year [3]. A previous pan-London audit identified that 4.6% of declined deceased donor organ offers were due to limitation in transplanting centre’s capacity to perform the operation. In late 2018, National Health Service Blood and Transplantation (NHSBT) asked the London transplanting units to form a collaborative to resolve local issues within kidney transplantation, address inequity in access to transplantation and build resilience. The Transplant Pan London Collaborative (Transplant PLC) set about standardizing organ offer declines across the five adult units and created a protocol for transferring both donor organ and transplant recipient to a neighbouring transplant unit, if the patient's designated unit was unable to perform their transplant in a timely fashion [4].

The aim of the sharing protocol was to enable transplantation into the intended (named) recipient, according to national allocation, when their own transplant unit was either unable to perform or could not perform the transplant in a reasonable timeframe, thus addressing inequity in transplantation.

This paper aims to describe the experiences of Transplant PLC in establishing a regional sharing protocol and evaluate the outcomes of patients transplanted under its auspices.

## MATERIALS AND METHODS

**Figure 1:**
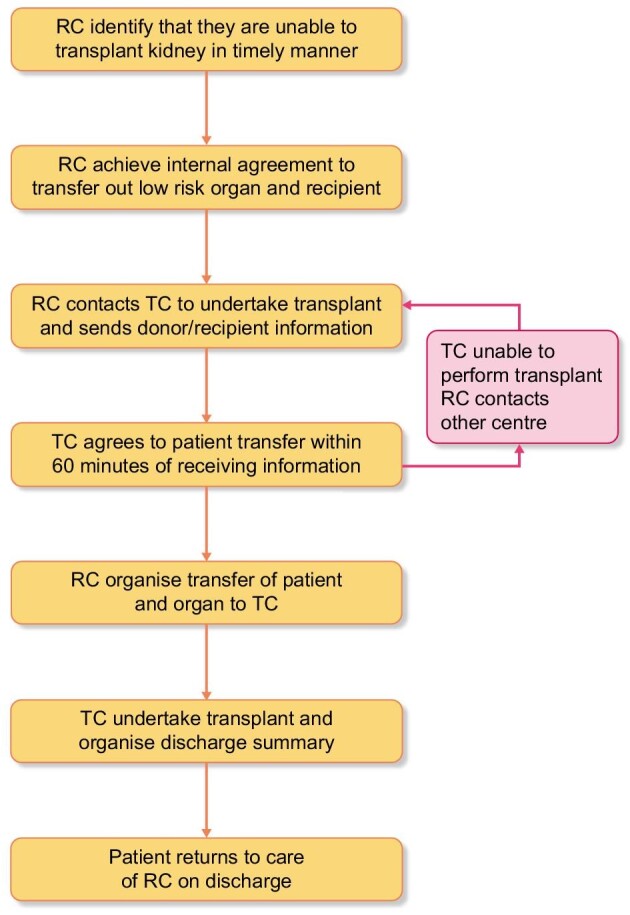
Organ-sharing protocol flowchart. The figure shows a flowchart outlining the sequence of events to initiate organ and recipient transfer between referring centre (RC) and transplanting centre (TC).

The Transplant PLC protocol provided a framework for resource and data sharing which enabled units to collaborate and overcome logistical limitations at a site that has accepted a deceased donor kidney offer for an intended recipient. The aim was to facilitate safe transfer of the recipient and allocated organ from the referring centre (RC) to the transplanting centre (TC), a site which had capacity to perform the transplant within the collaborative. All five units are completely independent of each other and able to perform at the same level of kidney transplant complexity. The guidance for the sequence of steps to initiate organ and recipient transfer is depicted in Fig. 1 [4].

In addition, patient information documentation was also developed to assist the teams involved in addressing patient queries and apprehensions regarding their surgery occurring at a different unit than they expected [4].

The protocol was initially devised for the transfer of low-risk donors and low-risk recipients. Standard criteria donors and first kidney transplant recipients with limited comorbidity were considered low risk. In addition, initially only standard immunological risk patients were considered [4]. Over time, however, the collaboration evolved to include higher-risk donors and recipients in addition to sensitized individuals.

When an RC identified that they were unable to implant a donor organ or implant within an acceptable cold ischaemia time (CIT) due to logistical constraints the protocol was initiated. The decision to utilize the protocol was made after reaching internal agreement between the on-call consultant surgeon and nephrologist at the RC. The intended transplant recipient was then asked whether they were prepared to receive their transplant at another London transplant unit. If the patient agreed to transfer, other units of Transplant PLC were simultaneously contacted through an encrypted messaging service and asked if they had capacity to transplant a potential anonymous recipient. Potential TCs able to facilitate the implant would contact the RC to have an initial anonymized discussion regarding the case with priority given in order of initial response. If a potential TC were prepared to implant the organ into the intended recipient, patient- and donor-specific details were shared between the two centres by secure channels under a previously signed data sharing agreement. The potential TC were given a maximum of 1 h to make a final decision on implant, with the available data and any further required information that was requested. If the potential TC were prepared to implant the organ, the organ and patient were transferred to the relevant centre. NHSBT was notified about the transfer of organ and patient to the potential TC. If the TC were unable to accept the referral, the next centre who had expressed interest would be contacted. Ultimately, if no TC was agreed the organ was declined and reallocated to another national centre.

The immunological risk assessment was undertaken in the RC, either virtually or with local serum samples. The recipient underwent transplantation at the TC under the defined protocols of that centre and was transferred back to the care of the RC for long term follow up, on discharge from hospital. The activity for the transplant was allocated to the TC while long term outcomes were allocated to the RC.

This was a retrospective observational cohort study on prospectively identified patients. All patients that underwent a deceased donor kidney transplant via the Transplant PLC scheme from 7 November 2020 to 29 July 2022 were included.

Data on kidney transplants performed via this collaborative scheme were obtained from NHSBT. NHSBT also supplied data regarding the occurrence of Simultaneous Pancreas-Kidney (SPK) transplants in participating centres (as a potential reason for centre capacity issues). Donor information was obtained from the NHSBT Organ Donation and Transplantation Electronic Offering System. This included cause of death, donor type and donor pre-terminal creatinine levels. The Organ Procurement and Transplantation Network (OPTN) calculator was used to calculate the Kidney Donor Profile Index (KDPI) and Kidney Donor Risk Index (KDRI) for all donor organs [5]. Expanded Criteria Donor was defined using the OPTN/United Network for Organ Sharing definition of all donors over age 60 years and donors aged 50–59 years with at least two of the three medical criteria: history of hypertension, terminal predonation creatinine >1.5 mg/dL (132.63 µmol/L) and death due to cerebrovascular accident [6].

Recipient data were obtained from electronic and paper records from TCs and RCs. This included demographic details, past medical history of transplantation and renal replacement therapy, perioperative details and length of stay, graft function and patient survival.

Creatinine levels at Day 7, and 3, 6 and 12 months post-transplantation were obtained for alive recipients. Creatinine levels for those on kidney replacement therapy (KRT) were excluded in analysis. Delayed graft function (DGF) was defined as any KRT within the first 7 days post-transplant.

Outcome data included creatinine levels as an indicator of graft function, length of stay, death and surgical post-operative complications. Percentages, median values and the interquartile range (IQR) were calculated using GraphPad Prism 10.2.3. Graft function and mortality was followed up over 12 months. Information regarding post-transplantation complications included the incidence of rejection, requirement for operative or radiological re-intervention over the initial 3-month follow up period was obtained.

This study was an agreed audit of clinical practice across the Transplant PLC units. We describe the activity and outcomes over the initial 20-month period, spanning November 2020 to July 2022.

## RESULTS

### Use of the scheme

A total of 16 recipients were transplanted between the 7 November 2020 and 29 July 2022 (20 months and 22 days). This represents 1.53% of the 1048 deceased donor kidney transplants performed across the five London transplant units during this period.

The reasons for referral to the scheme were insufficient theatre capacity (68.75%) and IT systems failure (31.25%).

No request for mutual aid was unanswered within the study period.

All five units transplanted recipients on the scheme. Four units referred recipients to the scheme.

### Recipient characteristics

Recipient characteristics are summarized in Table 1.

**Table 1:** Recipient characteristics.


Median age, years (IQR)	55 (21)
Median BMI, kg/m^2^ (IQR)	24.35 (4.93)
Median waiting time, days (IQR)	1075 (999.7)
Median time on dialysis, months (IQR)	28 (37.75)^a^
Female, *N* (%)	8 (50)
Transplant status, *N* (%)	
First transplant	12 (75)
Second transplant	4 (25)
Dialysis status, *N* (%)	
Haemodialysis	11 (68.75)
Peritoneal dialysis	3 (18.75)
Pre-dialysis	2 (12.5)
Calculated reaction frequency, *N* (%)	
0%	10 (62.5)
>0–<85%	3 (18.75)
≥85–100%	3 (18.75)
Crossmatch, *N* (%)	
Wet	10 (62.5)
Virtual	6 (37.5)
COVID-19 vaccination status, *N* (%)	
Vaccinated	13 (81.25)
Unvaccinated	3 (18.75)

^a^Two pre-emptive recipients analysed as 0 months on dialysis.

BMI, body mass index.

No recipient declined the offer of transfer to another unit for transplantation. The most common cause of end-stage kidney disease was hypertension (five recipients, 31.25%).

### Donor characteristics

Donor characteristics are summarized in Table 2.

**Table 2:** Donor characteristics.


Median age, years (IQR)	51 (24.5)
Median Kidney Donor Risk Index, score (IQR)	1.18 (0.588)
Median Kidney Donor Profile Index, score (IQR)	68 (46.25)
Median terminal pre-donation serum creatinine, µmol/L (IQR)	65.5 (51.3)
Donor type, *N* (%)	
Donation after brainstem death	10 (62.5)
Donation after circulatory death	6 (37.5)
Standard criteria donor	8 (50)
Expanded criteria donor	8 (50)
Acute kidney injury, *N* (%)	4 (25)
Received kidney replacement therapy, *N* (%)	1 (6.25)
Smoking history, *N* (%)	9 (56.25)
Diabetes, *N* (%)	2 (12.5)
Hypertension, *N* (%)	5 (31.3)

The most common cause of death amongst donors was intracranial haemorrhage (56.25%).

One donor had evidence of transmissible disease at time of offer (genital warts). One donor had a history of intravenous drug use with the cause of death being heroin overdose. One donor had malignancy (most likely glioblastoma, World Health Organisation grade 4 on biopsy with no evidence of metastatic spread).

All donor kidneys had single ureters. Half of the donor kidneys had single arteries.

### Intra operative course

The median CIT was 13 h 7 min (IQR 5 h 19.3 min). The median anastomosis time was 37.5 min (IQR 25.75 min). Three patients (18.75%) required arterial reconstruction. Two involved end-to-side anastomoses of a smaller artery onto a larger artery. One involved external iliac artery reconstruction that had been anticipated during the pre-transplant evaluation period. Intra-operative complications consisted of higher than standard blood loss (two cases), ostial avulsion of the main renal artery (one case) and re-do anastomosis due to a dusky kidney (one case). Twelve patients (75%) went to the standard post-operative care location. Four patients (25%) required higher levels of care. In two patients this was planned due to their medical history. The other two patients had unplanned high dependency unit admissions.

### Post-operative surgical complications

Seven patients (43.75%) experienced post-operative complications within the first 3 months of transplantation.

Post-operative complications are listed in Table 3.

**Table 3:** List of surgical complications encountered.

Details	Frequency
Catheterization for urinary retention	1
Perinephric haematoma managed conservatively	1
Radiologically guided drainage of perinephric collection	2
Transplant nephrostomy and ureteric stent insertion	1
Bleeding from anastomosis requiring surgical intervention	1
Bleeding from mesoureter requiring surgical intervention	1
Arterial thrombus requiring surgical intervention	1
Ureteric reimplantation	1

### Induction and maintenance immunosuppression

Fifteen patients (93.75%) received basiliximab for induction and tacrolimus and mycophenolate mofetil for maintenance. Thirteen of these patients remained on prednisolone at 3 months. One patient (6.25%) received alemtuzumab for induction with tacrolimus for maintenance.

### Post-operative graft function

Post-operative median creatinine levels in surviving non-dialysis dependent patients are summarized in Table 4.

**Table 4:** Post-operative median creatinine levels in surviving non-dialysis dependent patients.

Time after transplant	Median creatinine, µmol/L (IQR)	Number of patients, *N*^a^
7 days	270 (165)	14
3 months	121 (80)	15
6 months	143.5 (101.7)	14
12 months	134 (70)	11

^a^Dialysis-dependent patients due to DGF or graft failure and deceased patients have been excluded.

Seven patients (43.75%) had DGF. There were no cases of primary non-function. At 3 months post-transplant no patients were receiving dialysis. Two graft failures occurred in the 13 surviving patients within the 12-month follow-up period. One was due to rejection and infection at 239 days post-transplant. The other had poor graft function with return to dialysis at 394 days post-transplant.

### Biopsy and Rejection

Nine recipients (56.3%) had a for-cause biopsy within the first 3 months. One recipient had rejection. This was antibody mediated.

### Length of stay

The median length of stay was 7.5 days (IQR 9.25 days).

One patient was repatriated to their referring centre for inpatient rehabilitation. All other patients were discharged from the transplanting centre to their place of abode.

### Mortality and causes of death within the first post-operative year

Three deaths (18.8%) occurred in the first 12 months post-transplant. Two patients died due to COVID-19 (at 71 and 160 days post-transplant). Both these patients had been vaccinated prior to their transplant. One patient died 162 days post-transplant due to a likely cardiac event.

## DISCUSSION

The maintenance of access to transplantation is a key priority in providing equity for all transplant recipients. Despite the benefit of a national system of organ allocation, transplant recipients may occasionally miss out on the opportunity of transplantation when there is insufficient local resource to undertake their transplant. The fluctuating nature of organ allocation with peaks in activity or the occurrence of major incidents can occasionally reduce capacity within a transplant unit and therefore limit the equity of access to transplantation.

The main reasons for reduced capacity identified in our study period resulting in referral to the scheme were insufficient theatre capacity and IT systems failure.

Insufficient theatre capacity occurs due to the lack of guaranteed immediate access to theatre when there is a kidney to be implanted. This can be due to multiple specialties competing for limited emergency theatre capacity or due to other activity being undertaken by RC transplant team. Multiple organs may be due to arrive at a transplant unit in a short space of time due to the unpredictable nature of the national allocation system. This could result in unacceptably long CIT. In 5 of 11 (45.45%) instances of insufficient theatre capacity a simultaneous pancreas–kidney transplant was occurring at the RC. Two of the five units in the collaborative perform pancreas transplantation and this lengthy procedure has the potential to significantly delay other patients awaiting transplantation.

The IT systems failure occurred at one unit spanning a period of 9 days [7]. This was due to temperatures reaching 40°C for the first time in the UK resulting in data centre failures due to overheating [8]. Organ offers accepted by this unit during this period were therefore referred to the scheme.

Our local collaborative provided a protocol to establish mutual aid between regional transplant units in London and utilize additional capacity within a neighbouring transplant unit. The sharing protocol facilitated 16 nationally allocated transplant procedures to go ahead where they would have otherwise been declined due to capacity issues within the allocated centre. This system enabled these recipients to receive the kidney that they had been allocated within the UK program and restore equity of access to transplantation. Differing practice between the five units involved in the collaboration, with regards to immunosuppression protocols was managed through clear communication. Units performing the transplant via the protocol were renumerated for the procedure without outcomes being allocated to the referring unit.

Feasibility of organ sharing networks in the UK is not a novel idea. The Coventry-Oxford Network for Transplantation (COxNeT) [9, 10] has already shown that partnership between two units can enable mutual aid. However, unlike COxNet where there is systemic integration between these two units, the five units in the Transplant PLC are completely independent of each other, making this collaboration more challenging and thus more impressive in its implementation. In addition to mutual aid, the collaboration has also looked at standardizing pre-transplantation work-up and auditing outcomes.

The Transplant PLC scheme initially functioned on a case-by-case basis with referrals due to limited theatre capacity. It subsequently accommodated multiple referrals over a short space of time in the setting of a major IT systems failure at a single unit. Although initially developed for low-risk donors and recipients, the scheme progressed to transplantation in medically and surgically complex recipients with organs from extended criteria donors. Timely transfer of data and communication between multiple medical and surgical teams enabled complex transplants to occur; for example, the instance of a patient who had a planned iliac artery reconstruction with polyester graft at the time of transplant or transplantation in a patient with homozygous sickle cell disease requiring pre-operative transfusion.

In this small sample, 3 (18.75%) of the 16 patients did not survive their first transplant year. Two patients died due to COVID-19 infection in March 2022 and December 2022. Neither recipient contracted COVID-19 during their transplant index admission, and it is equally likely that they may have contracted COVID-19 in the community if transplanted in their usual centre. One patient died due to cardiac causes 5 months post-transplant. This transplant recipient was known to be high risk, with previous cardiac stenting however having been medically maximized prior to transplantation.

With regards to the two patients who died due to COVID-19, both were vaccinated prior to transplant but died during the Omicron wave of late 2021/early 2022, prior to the availability of the booster covering omicron variants. The reported mortality of COVID-19 among a cohort of kidney transplant recipients within the first year of transplant at one of the participating units was 2.5% [11].

It is challenging to compare national rates of recognized early surgical post-operative complications such as re-exploration and drainage of perinephric collections with this small sample size. However, the experience described highlights the ability of the participating units to manage such events in a heterogeneous group of transplant recipients (including 25% patients having had one prior kidney transplant).

The median length of stay of 7.5 days (range 4–75 days) is comparable with nationally reported figures of 5–7 days in units using Enhanced Recovery After Surgery (ERAS) pathways in the 2021 survey of all UK transplant units [12].

Overall, the Transplant PLC activity reported above demonstrates the feasibility of a multi-site collective resource sharing kidney transplantation scheme that allows the provision of mutual aid and restoration of equity of access to transplantation. Key to its success has been good pre-existing and continued development of relations and fostering of trust between the five transplant units in London. Longer term follow-up data on graft and patient survival, prospective expanded data collection for future use of the protocol would be of benefit, along with qualitative evaluation of the recipient's perspective to optimize the patients’ experience of the scheme.

## Data Availability

The data underlying this article will be shared on reasonable request to the corresponding author provided necessary permissions are obtained from the NHS trusts, and patient and donor confidentiality is maintained.

## References

[1] POL186/12–Kidney Transplantation: Deceased Donor Organ Allocation [Internet]. NHS Blood and Transplant; 2022.

[2] ODT Clinical—NHS Blood and Transplant [Internet]; [cited 2024 Aug 22]. National Organ Retrieval Services. Available from: https://www.odt.nhs.uk/retrieval/national-organ-retrieval-services/ (14 April 2024, date last accessed).

[3] Organ and Tissue Donation and Transplantation Activity Report 2022/23 [Internet]. NHS Blood and Transplant; 2023 [cited 2024 Apr 9]. Available from: https://nhsbtdbe.blob.core.windows.net/umbraco-assets-corp/30198/activity-report-2022-2023-final.pdf (14 April 2024, date last accessed).

[4] Transplant: Pan London Collaborative Organ Sharing Protocol [Internet]. NHS Blood and Transplant; 2019. Available from: https://nhsbtdbe.blob.core.windows.net/umbraco-assets-corp/17468/transplant-plc-sharing-protocol-20191129.pdf (14 April 2024, date last accessed).

[5] KDPI calculator—OPTN [Internet]; [cited 2024 Apr 9]. Available from: https://optn.transplant.hrsa.gov/data/allocation-calculators/kdpi-calculator (14 April 2024, date last accessed).

[6] Metzger RA, Delmonico FL, Feng S et al. Expanded criteria donors for kidney transplantation. Am J Transplant 2003;3:114–25. 10.1034/j.1600-6143.3.s4.11.x 12694055

[7] Guy's and St Thomas’ NHS Foundation Trust . Review of the Guy's and St Thomas’ IT critical incident final report from Deputy Chief Executive Officer January 2023 [Internet]. 2023; [cited 2024 Aug 22]. Available from: https://www.guysandstthomas.nhs.uk/sites/default/files/2023-01/IT-critical-incident-review.pdf (14 April 2024, date last accessed).

[8] Heatwave: Fires blaze after UK passes 40C for first time. BBC News [Internet]. 2022; Jul 19 [cited 2024 Aug 22]. Available from: https://www.bbc.com/news/uk-62217282 (14 April 2024, date last accessed).

[9] Oxford University Hospitals NHS Foundation Trust. National transplant award for Coventry-Oxford Network for Transplantation—Oxford University Hospitals [Internet]; [cited 2024 Apr 9]. Available from: https://www.ouh.nhs.uk/news/article.aspx?id=1893&pi=0 (14 April 2024, date last accessed).

[10] National award success for Coventry-Oxford Network for Transplantation [Internet]; [cited 2024 Apr 9]. Available from: https://www.uhcw.nhs.uk/news/national-award-success-for-coventry-oxford-network-for-transplantation/ (14 April 2024, date last accessed).

[11] Clarke C, Lucisano G, Prendecki M et al. Informing the risk of kidney transplantation versus remaining on the waitlist in the coronavirus disease 2019 era. Kidney Int Rep 2020;6:46–55. 10.1016/j.ekir.2020.10.032 33173838 PMC7644242

[12] Amer A, Scuffell C, Dowen F et al. A national survey on enhanced recovery for renal transplant recipients: current practices and trends in the UK. Ann R Coll Surg Engl 2023;105:166–72. 10.1308/rcsann.2021.0365 35446720 PMC9889185

